# Simulating an Integrated Photonic Image Classifier for Diffractive Neural Networks

**DOI:** 10.3390/mi15010050

**Published:** 2023-12-26

**Authors:** Huayi Sheng, Muhammad Shemyal Nisar

**Affiliations:** Sino-British College, University of Shanghai for Science and Technology, Shanghai 200093, China; hs1223@ic.ac.uk

**Keywords:** integrated photonics, computing metasurfaces, diffractive neural networks, photonic image classifier

## Abstract

The slowdown of Moore’s law and the existence of the “von Neumann bottleneck” has led to electronic-based computing systems under von Neumann’s architecture being unable to meet the fast-growing demand for artificial intelligence computing. However, all-optical diffractive neural networks provide a possible solution to this challenge. They can outperform conventional silicon-based electronic neural networks due to the significantly higher speed of the propagation of optical signals (≈ $10^{8}$ m.s ${\text{​}}^{-1}$ ) compared to electrical signals (≈ $10^{5}$ m.s ${\text{​}}^{-1}$ ), their parallelism in nature, and their low power consumption. The integrated diffractive deep neural network (ID ${\text{​}}^{2}$ NN) uses an on-chip fully passive photonic approach to achieve the functionality of neural networks (matrix–vector operations) and can be fabricated via the CMOS process, which is technologically more amenable to implementing an artificial intelligence processor. In this paper, we present a detailed design framework for the integrated diffractive deep neural network and corresponding silicon-on-insulator integration implementation through Python-based simulations. The performance of our proposed ID ${\text{​}}^{2}$ NN was evaluated by solving image classification problems using the MNIST dataset.

## 1. Introduction

Neural networks have long been in use for a variety of tasks, including but not limited to pattern recognition [1], speech recognition [2], image classification [3], anomaly [4] and defect detection [5], and natural language processing [6]. Neural networks are computational models that were inspired by the brain and thus tend to emulate the working of the brain. Interconnected nodes or neurons (as they are referred to in the literature) are organized into layers, and each neuron receives an input signal, performs a computation on it, and provides an output that is in turn fed as an input into the next layer. This architecture, along with the application of back-propagation methods [7], enables the learning of the neurons, which can subsequently be used for the computation of unseen data. While this architecture has enabled remarkable improvements in computational power, it has recently reached a bottleneck due to the increased complexity and volume of data. One of the important bottlenecks, in this case, is the increasing amount of computational resources required for such neural networks, leading to exorbitant power consumption [8] to keep them operative and the need for improved speed [9,10].

There have been numerous solutions to the problem of increasing power consumption [11,12,13,14], but few of them can rectify it as effectively as a photonic neural network [10]. Taking this lead, many research groups have tried to exploit the potential of photonics and have assembled low-power passive photonic networks to realize tasks such as image classification [15,16]. The earliest conception of optical and photonic neural networks can be traced back to studies such as that presented by Stoll and Lee in 1984 [17] and that of Abu-Mostafa [18]. These were very primitive devices; nevertheless, they were an important development that subsequently resulted in advances such as photonic reservoir computing [19,20], various bioinspired and neuromorphic computers [21,22], and finally photonic neural networks [23,24,25]. The modern conceptions of photonic neural network implementation can be categorized into two groups: (i) photonic neurons and synapses [26,27] and (ii) diffractive neural networks [23,24,25]. While both methods have their own merits, the photonic neuron- and synapse-based methodology shows only limited non-linearity [28], is sensitive to input variations [29], and has limited scalability [30]. This makes diffractive neural networks an interesting alternative to be explored for the implementation of machine learning applications.

All-optical diffractive neural networks have shown promising results, but most of these implementations are based on free-space optics [10,15,28], which, although the principles are valid, is of little utility for actual computational platforms. On the other hand, there have been some recent reports of integrated photonics platforms implementing image classification on integrated platforms [23,25]. However, they present varying degrees of success. The pioneering work on this subject was carried out by Zi Wang et al. [23], who were able to implement an integrated metasystem to perform image processing. While they demonstrated Python implementation using MNIST, the physical implementation was carried out on a dataset with images of an even lower resolution. This shows that the practical implementation of a neural network with a large number of pixels is extremely difficult, making this characteristic a limitation in the practical implementation of such neural networks. This is a significant limitation that should be addressed if diffractive neural networks are to be of any practical use in real-world applications.

**Figure 1:**
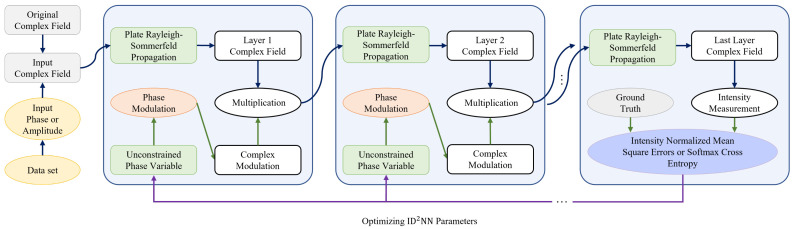
Design framework of the integrated diffractive deep neural network.

Silicon-based diffractive neural networks face numerous challenges, such as the increased complexity of training due to the complex nature of the diffractive network, limited precision, and cumbersome physical implementation. To address some of these limitations in the practical implementation of diffractive neural networks, in this paper, we present a simplified Python-based neural network (Figure 1). We believe that this methodology could improve the operation of all-optical photonic neural networks and pave the way for improved integrated devices.

## 2. Design Framework of Integrated Diffractive Deep Neural Network

The integrated diffractive deep neural network was designed based on a fully connected system architecture [15,31]. The attributes of the dataset can be encoded into the amplitude channel or phase channel of the original complex field to obtain a new input complex field. The resulting complex field of slab waveguide propagation on the plate is multiplied by a phase modulator at each layer and is then transferred to the next layer. To optimize the neural values in the ID ${\text{​}}^{2}$ NN, an error back-propagation algorithm is used, which is based on the loss function of normalized softmax cross-entropy, measuring the distance between the desired target intensity and the real output intensity in the detector plane. The trained neuron values are then mapped onto the different phase delays in terms of physical structures, and the corresponding phase delays are produced by varying the length of the etched slots. Each neuron value is approximated by one slot [16]. After this mapping process, the ID ${\text{​}}^{2}$ NN design is fixed; once manufactured by electron-beam lithography and other microelectronic processes, it performs the learned function at the speed of light.

### 2.1. Physical Input to the Integrated Diffractive Deep Neural Network

In this work, the pixel values were converted into binary format (0/1) to simulate light transmission and blocking, endowing the binarized input with actual physical contour information. This procedure relied on micro–nano manufacturing techniques to create tangible input entities. A pixel grid with a value of 1 represents the presence of light, which will propagate forward as a light source through diffraction, thus enabling information to be loaded in the amplitude channel.

**Figure 2:**
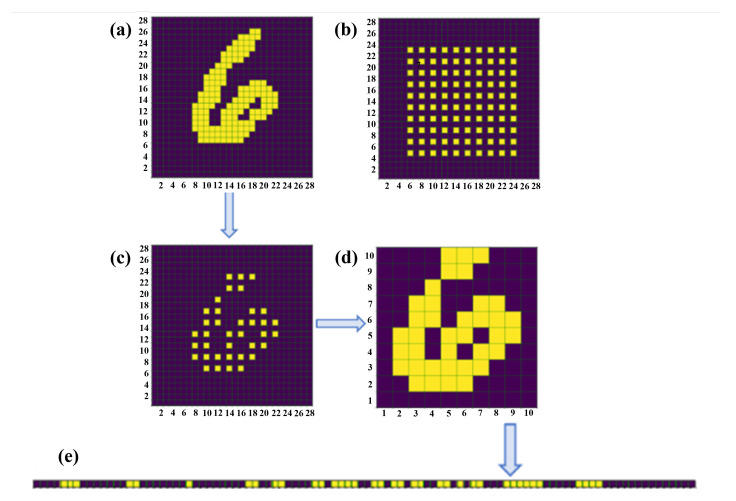
Amplitude channel encoding of input field: (**a**) original image with default resolution (28 × 28), (**b**) selected pixels shown with light squares, (**c**) selected pixels in the original image, (**d**) downsampled image with 10 × 10 pixels, (**e**) flattened image (1 × 100).

To minimize power consumption and area overhead, non-essential characteristics for image classification were removed. Each pixel within the training image served as a feature for the ID ${\text{​}}^{2}$ NN. The dataset was downscaled to N≤784 features, with *N* ranging from 36 to 324, in order to determine the optimal number of observed features. Classification accuracy was obtained for the downsampled data in Python simulations of the ID ${\text{​}}^{2}$ NN. The optimal number of observed features was N=100 (10 × 10), which corresponded to a 91.6% accuracy for the MNIST dataset. Figure 2 shows the binarized and downsampled N=100 image. Flattening was necessary for the functioning of the integrated diffractive deep neural network in a two-dimensional plane.

### 2.2. Forward Propagation and Error Back-Propagation Model

For a guided wave system, the wavelength of light inside the medium depends on the refractive index of the medium: $\lambda_{g}$ = λ/n , where λ is the free-space wavelength, *n* is the refractive index of the guided material, and $\lambda_{g}$ is the wavelength in that material. According to the Rayleigh–Sommerfeld diffraction equation [32], when waves propagate to the position ( $x_{i}$ , $y_{i}$ , $z_{i}$ ) in the *l*th layer, the following optical field will be generated at point *i* located at (*x*,*y*,*z*) in the next layer [23]:

(1) $$w_{i}^{l}\left(x,y,z\right)=\frac{z-z_{i}}{r^{2}}\left(\frac{1}{2\pi r}+\frac{1}{j\lambda_{g}}\right)e^{\frac{j2\pi r}{\lambda_{g}}}$$

where $w_{i}^{l}$ is the propagation coefficient, $\lambda_{g}$ is the wavelength of the optical field, and $r=\sqrt{{\left(x-x_{i}\right)}^{2}+{\left(y-y_{i}\right)}^{2}+{\left(z-z_{i}\right)}^{2}}$ is the Euclidean distance between the two points. Thus, the output of the neuron at point ( $x_{i}$ , $y_{i}$ , $z_{i}$ ) in the *l*th layer is the sum of the outputs of all neurons at the position (*x*,*y*,*z*) in the ( l−1 )th layer multiplied by the complex transmission coefficient for that neuron at that point and $w_{i}^{l}$ (*x*,*y*,*z*):

(2) $$n_{i}^{l}\left(x,y,z\right)=w_{i}^{l}\left(x,y,z\right).t_{i}^{l}\left(x_{i},y_{i},z_{i}\right).\sum_{k}n_{k}^{l-1}\left(x_{i},y_{i},z_{i}\right)$$

The forward propagation process of the ID ${\text{​}}^{2}$ NN can therefore be summarized as follows:

(3) $$\begin{array}{cc} \; & n_{i}^{l}\left(x,y,z\right)=w_{i}^{l}\left(x,y,z\right)\cdot t_{i}^{l}\left(x_{i},y_{i},z_{i}\right)\cdot m_{i}^{l}\left(x_{i},y_{i},z_{i}\right)\\ \; & m_{i}^{l}\left(x_{i},y_{i},z_{i}\right)=\sum_{k}n_{k}^{l-1}\left(x_{i},y_{i},z_{i}\right)\\ \; & t_{i}^{l}\left(x_{i},y_{i},z_{i}\right)=\alpha_{i}^{l}\left(x_{i},y_{i},z_{i}\right)\cdot e^{j\phi_{i}^{l}\left(x_{i},y_{i},z_{i}\right)} \end{array}$$

The two latent variables $\alpha_{i}^{l}$ and $\phi_{i}^{l}$ should be confined to the ranges (0, 1) and (0, 2π ), respectively. Equation (3) represents a recursive model of the forward propagation process of the ID ${\text{​}}^{2}$ NN. Suppose that the entire ID ${\text{​}}^{2}$ NN consists of M layers (M≥l≥1) , and in order for that model to hold, it is necessary to know the initial value of the entire recursive relationship, that is, the value of $\sum_{k}n_{k}^{0}\left(x_{i},y_{i},z_{i}\right)$ when M=l=1 . In fact, the input layer is the 0th layer of the ID ${\text{​}}^{2}$ NN, and the encoded optical field carrying the physical information is that initial value. The (M+1) th layer is the output layer, in which the detectors measure the intensity of the resulting field in predefined regions:

(4) $$s_{i}^{M+1}={\left|m_{i}^{M+1}\right|}^{2}$$

To utilize the error back-propagation algorithm, normalized softmax cross-entropy (NSCE) is used. For the N-class image classification task with N detection regions included, the normalized intensity distribution $\tilde{s_{n}}$ of the *n*th detector region can be calculated as $\tilde{s_{n}}=\frac{s_{n}}{maxs_{n}}$ . $\tilde{g_{n}}$ is defined as the expected normalized field intensity. Based on the NSCE loss function, the optimization problem for an ID ${\text{​}}^{2}$ NN design can be written as

(5) $$\min_{\phi_{i}^{l}}\left(-\sum_{n=1}^{N}\tilde{g_{n}}\log\left(\frac{\exp(\tilde{s_{n}})}{\sum_{n=1}^{N}\exp\left(\tilde{s_{n}}\right)}\right)\right),\;0\leq \phi_{i}^{l}\leq 2\pi$$

The weights of each diffractive layer can be iteratively updated through backpropagation algorithms so that the loss will continuously drop-down, meaning that the actual intensity distribution is approaching the desired result. To update the weights in the network through a gradient descent optimization process, the gradient of the loss function with respect to the training variables, the gradient can be derived as:

(6) $$\frac{\partial E(\phi_{i}^{l})}{\partial \phi_{i}^{l}}=\frac{\partial E(\phi_{i}^{l})}{\partial {\left|m_{i}^{\left(M+1\right)}\right|}^{2}}\cdot \frac{\partial {\left|m_{i}^{\left(M+1\right)}\right|}^{2}}{\partial \phi_{i}^{l}}=\left(\left({\hat{s}}_{n}-{\tilde{g}}_{n}\right)\cdot 2\cdot \mathrm{real}\left\{{\left(m_{i}^{\left(M+1\right)}\right)}^{*}\cdot \frac{\partial m_{i}^{\left(M+1\right)}}{\partial \phi_{i}^{l}}\right\}\right)$$

### 2.3. Neural Value Mapping and Verification of the Designed ID ${\text{​}}^{2}$ NN

**Figure 3:**
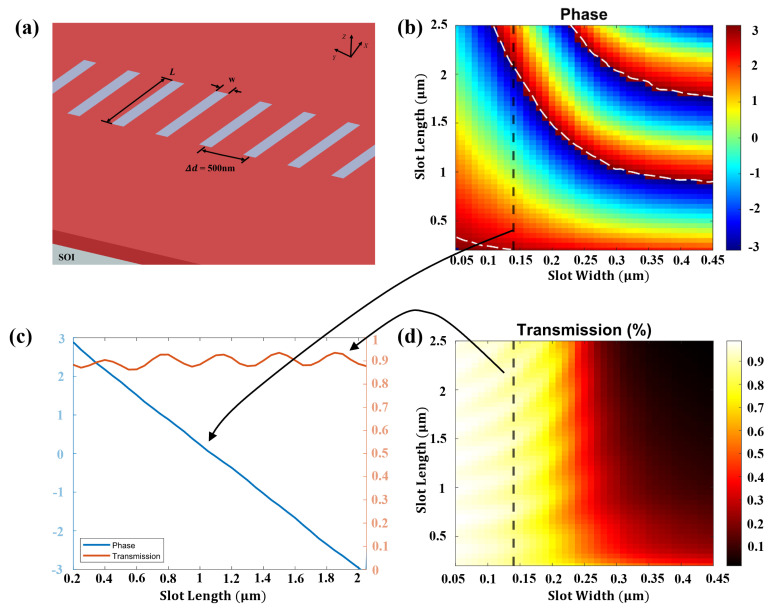
Neuron value mapping. (**a**) Schematic view of unit cells (slots) in an integrated diffraction layer with a feature size Δd=500 nm, defined on the SOI substrate. This could impose a localized phase shift on the optical wave traveling in plane. (**b**) and (**d**) respectively are the simulated phase and amplitude retardation of the transmission as a function of the slot width and length. (**c**) Neural value mapping relationship when the input light wavelength was 1550 nm and the slot width was fixed to be 140 µm.

The term “neuron value mapping” in designing an ID ${\text{​}}^{2}$ NN refers to using phase delays imposed on the optical field through slots of different lengths. The phase delays were obtained from the training in the computer, as discussed in the previous section. The designed unit cells were rectangular etched arrays created in the silicon-on-insulator (SOI) structure, as shown in Figure 3a. The silicon membrane of the SOI structure had a thickness of 250 nm, while the SiO ${\text{​}}_{2}$ insulator layer was 2 µm thick. The feature size of the unit cell ( δd ) was set to be 500 nm, which was less than half of the wavelength. The large contrast in refractive index between silicon and silicon dioxide enabled a 0 to 2π phase delay while maintaining a high transmission. The phase delay/phase shift and transmission could be controlled by varying the width and length of the slot. The simulation results of the phase and amplitude retardation of transmission as a function of both the slot’s width and length are shown in Figure 3b,d. By maintaining the slot width at 0.14 µm and varying the slot length from 0.2 to 2.5 µm, a phase shift from 0 to 2π and a transmission greater than 90% could be simultaneously achieved.

To numerically demonstrate the performance of the designed integrated diffractive deep neural network, we employed the prototypical machine learning task of image classification using the MNIST dataset, including the downsampling and loading processes. One hundred binarized inputs were loaded onto the corresponding narrow input waveguides in the amplitude channel, and the classified results comprised ten categories, ranging from 0 to 9. The structural parameters across the entire ID ${\text{​}}^{2}$ NN system are shown in Table 1.

## 3. Results and Discussion

**Figure 4:**
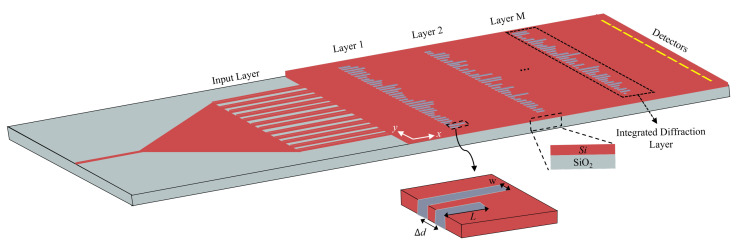
Schematic of the integrated diffractive deep neural network showing a single input connected to the taper that feeds into the input layer.

The system set up with these parameters was initially established to learn where the inputs were loaded onto the waveguides in a similar manner to the weights on the neural network shown in Figure 4. The earlier work of Wang et al. [23] used an array of grating couplers to provide the input, but this introduced losses in the input, as no grating couplers are lossless [33,34], and increased the footprint of the device by many folds. These are the major considerations that led [23] to the adoption of a different dataset for the actual fabricated device.

Due to these considerations, the method adopted in this study involved the inputs being hardcoded in the photonic crystal. By hardcoding, we mean that the input layer of the neural network shown in the schematic of Figure 4 was arranged in such a way that a waveguide was added to the location where the pixel value was “1”, while there was no silicon waveguide at the location where the pixel value was “0”, thus reducing the signal output from that location to zero. This method is referred to as hardcoding because the input, once encoded into the photonic crystal, cannot be changed. This also implies a separate photonic crystal containing the neural network for each input. It is essentially similar to the method adopted in other similar studies [16].

However, contrary to these studies, the proposed design reduced the device footprint compared to the use of grating couplers [23], providing a more compact and manageable device that enables the extension of the system to an increased number of inputs. At the moment, the proposed design suffers from input inflexibility due to the inputs being hardcoded, as they cannot be changed post-fabrication. However, the proposed design could be implemented with phase-change materials in the future to better incorporate programable inputs into the photonic neural network platform. A possible future development of such an integrated chip in a photonic neural network using phase-change materials was presented by Nisar et al. [35]. While phase-change materials are already being used for numerous applications including in-memory computing [36] and other memory devices [37], their use in photonic neural networks could increase these networks’ vitality.

**Figure 5:**
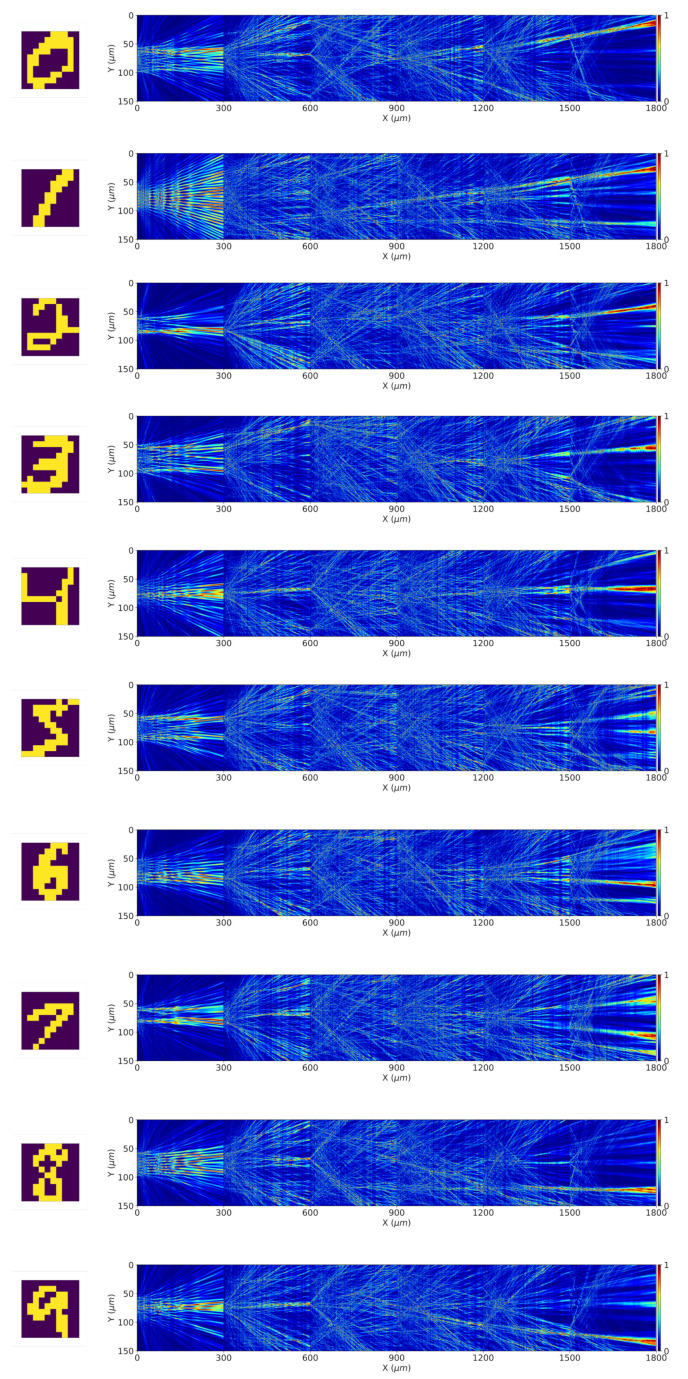
From top to bottom, the inputs were handwritten digits from 0 to 9 (shown in the left column), and their corresponding outputs from different layers of the diffractive neural network are shown, with the final output at the right end of each diagram. The integrated diffractive deep neural network could accurately perform the classification task for handwritten digits.

The simulation and training of such large-scale optical devices with small variations using expensive licensed conventional photonic software like Lumerical FDTD would require significant amounts of computational and financial resources over a substantial length of time. To optimize the resource allocation and overcome financial constraints, we used the Rayleigh–Sommerfeld diffraction equations given above and trained the weights of the neural network on Python with the MNIST handwriting dataset. The optimized neural network was able to achieve 91.6% accuracy. These results are shown in Figure 5. There was a loss of 0.46 dB per diffraction layer at the operating wavelength of 1550 nm. This was achieved through a device that was optimized to operate with 1500 neurons divided among five layers of 300 neurons each. The resulting device had an approximate footprint of 282,000 m ${\text{​}}^{2}$ .

In order to train the neural network, the length of each slot representing a neuron was varied in accordance with the results shown in Figure 3 to achieve the optimized output. After the process of optimization, final optimized values were achieved for each of the 1500 neurons that would recognize the input encoded at the input layer. Figure 5 shows typical outputs from this dataset for each input in the left column. This input was encoded in the input layer, at the “0” mark on the x-axis. Subsequently, the optical waves from the input layer traversed all five layers until they reached the output plane shown at the right end of the image. The output plane contained detectors to detect the incoming waves in such a way that the receivers for each of the numbers 0 to 9 were arranged in the ascending order from top to bottom. At the output plane in the Figure 5, it can be observed that the optical waves were able to achieve constructive interference at the spot designated for that number at the input.

Following the training and testing of the neural network on Python, the system could ultimately also be verified on optical simulation suites. For this purpose, the system simulation design can be set up as shown in Figure 4.

## 4. Conclusions

This study devised an innovative method to provide inputs to a photonic neural network, contrasting with earlier studies that used grating couplers, resulting in an increased footprint for the device. Our method produced photonic neural networks with a smaller footprint as we did not include grating couplers, which also simplified the fabrication and testing of the devices with larger inputs as opposed to testing on much smaller images.

Although at the current stage the proposed device presents post-fabrication inflexibility, as the inputs are hardcoded in the photonic crystal, our work lays the foundations for future enhancements.The most promising of these is the enabling of programable inputs through the integration of phase-change materials. The application of such materials, as indicated by Nisar et al. [35], has showcased remarkable potential in various computing and memory devices, hinting at its potential to vitalize photonic neural networks.

In our design, we proposed a five-layer ID ${\text{​}}^{2}$ NN with 300 neurons in each layer. The whole system contains 1500 trainable neurons and showed a downsampled MNIST classification accuracy of 91.6%, which is comparable to state-of-the-art approaches.

The main losses in the ID ${\text{​}}^{2}$ NN include the propagation loss and transmission loss of the diffraction layer. The loss per integrated diffraction layer was less than 0.46 dB at a wavelength of 1.55 µm. For the scale of the proposed system, the total footprint was approximately 2.7 mm ${\text{​}}^{2}$ , which is smaller than the previously presented MZI-based photonic neural network. In summary, we put forward an on-chip photonic ID ${\text{​}}^{2}$ NN architecture based on a SOI structure that could perform complicated functions, providing a solution to address the growing needs of artificial intelligence systems.

This study successfully simulated, trained, and tested our optimized neural network using Python, demonstrating the convergence of optical and computational approaches. This successful undertaking lays the foundation for further optical simulation suite verifications and validations, leading to the thorough validation loop that is necessary to determine the effectiveness and functioning of the suggested system.

This study sets the stage for the potential intensive application of photonic neural networks in the future. This could be achieved by harnessing the full potential of photonics, phase-change materials, and optimized photonic crystals to design a compact, reprogramable platform that operates at the speed of light.

## Figures and Tables

**Table 1:** System design parameters of ID ${\text{​}}^{2}$ NN.

System Design Parameter	Parameter Value
Wavelength in free space	$1.55\times 10^{-6}$ m
Number of neurons per layer	300
Neuron feature size	$0.5\times 10^{-6}$ m
Distance between diffraction layers	$300\times 10^{-6}$ m
Number of hidden layers	5
Refractive index of diffraction layer	1.0
Refractive index of propagation medium	3.45

## Data Availability

Data are contained within the article.
